# Inhibitory effect of 17β-estradiol on the THIK-1 channel

**DOI:** 10.1371/journal.pone.0353757

**Published:** 2026-07-17

**Authors:** Michihiro Tateyama, Yoshihiro Kubo

**Affiliations:** 1 Division of Biophysics & Neurobiology, Department of Molecular and Cellular Physiology, National Institute for Physiological Sciences, Okazaki, Japan; 2 Physiological Sciences Program, Graduate Institute for Advanced Studies, SOKENDAI, Hayama, Japan; Indiana University School of Medicine, UNITED STATES OF AMERICA

## Abstract

A two-pore domain K^+^ (K2P) channel, THIK-1, plays important roles in microglia and macrophage. THIK-1 is known to be activated by arachidonic acid and G protein-coupled receptors and inhibited by anesthetics. Steroids, such as cholesterol, estradiol and progesterone, are known to modulate several K^+^ channels and they might be potential modulators of THIK-1. We examined the effects of steroids on THIK-1 and found that estradiol inhibits mouse THIK-1 by approximately 40% (IC_50_ = 4.9 ± 1.5 μM). Docking simulations of THIK-1 with estradiol indicated possible docking sites, which were further assessed by introducing an alanine mutation into a residue at or near these locations. The F142A, V269A, and Y273A mutations reduced the inhibitory effect of estradiol. These residues are situated within the upper cavity above the Y gate in THIK-1 (pond), suggesting that the pond conformation is essential for estradiol-mediated inhibition. Conversely, the F145A and F276A mutations, located outside this region, were inhibited by 10 nM estradiol and enhanced inhibition by estradiol, estrone, estriol, and progesterone, likely due to conformational changes that facilitate steroid inhibition. The mouse T237S mutation, which corresponds to the reported human THIK-1 variant, produced effects similar to those seen with the F145A and F276A mutations, but to a lesser degree. In summary, estradiol-mediated THIK-1 inhibition depends on residues located in the pond, which may have physiological or pathological significance for THIK-1 variants which are inhibited by low concentration of estradiol.

## Introduction

Two-pore domain K^+^ (K2P) channels possess two pore domains and four transmembrane (TM) domains, which form homo- or hetero-dimer as the functional K^+^ channel. The activity of K2P is modulated by changes in the physical and chemical environmental conditions, which are regulatory mechanisms of cellular functions [1,2]. A member of K2P, THIK-1 (*KCNK13*), is activated by arachidonic acid and inhibited by halothane [3]. The activity of THIK-1 channel is also regulated by anionic lipids [4], Gi/o-coupled receptors (Gi/o-Rs) [5–7] and Gq-coupled receptors (Gq-Rs) [7,8]. The studies of the THIK-1 knockout mice have demonstrated that the channel is critical for the function of microglia [6] and macrophage [9], and for the development of neuronal circuits [10]. THIK-1 is also suggested to be involved in an apoptosis process [11] and the development of Alzheimer’s disease and Parkinson’s disease [12]. Drugs that selectively target THIK-1 may help improvement of pathophysiological conditions [12–14].

THIK-1 is expressed in testis [3], which produces a steroid, testosterone. Steroids, such as cholesterol, progesterone and 17β-estradiol (estradiol), are known to regulate the activity of K^+^ channel through the direct interaction. Cholesterol activates KIR3.4 through interacting with the TM domain [15]. Cholesterol was also shown to interact with the lower TM domains of TASK-1 and TASK-3 [16,17] and the interaction was assumed to maintain the basal channel activity. Progesterone activates β1-containing BK channel possibly through interacting with the TM domain [18]. Another steroid, estradiol was reported to modulate the activity of the Maxi-K^+^ channel, the voltage gated K^+^ channels in the medial preoptic nucleus and the complex of Kv7.1 and NCNE1 [19–21]. The results suggest that THIK-1 may have the capacity to interact with steroid compounds. Here we examined the effects of several steroids on THIK-1. Among the tested steroids, estradiol showed the most potent inhibitory effect on THIK-1 but not on TREK-1, TRAAK and TASK-1. We further investigated important residues for estradiol-mediated inhibition by individually introducing alanine mutations to residues at or around the possible docking sites obtained from the results of the SwissDock simulation.

## Materials and methods

### Constructs, expression system and materials

The cDNA for mouse THIK-1 was subcloned into pcDNA3.1 (-) vector. The cDNAs of the coding region of TASK-1 and TREK-1 were isolated from mouse brain library and subcloned into pcDNA3.1 (-) vector. In this study, N-terminal 29 residues of mouse THIK-2 were deleted (ΔN-THIK-2), to increase surface expression [7,22]. The cDNAs for the human TRAAK channels were obtained from SinoBiological (Kanagawa, Japan). Coding regions of the mouse Gq-coupled adrenergic receptor α1A (α1A-AR, Met1-Arg368) with XhoI and BamHI restriction sites were amplified by the conventional PCR with designed primers, and the resulting products were cut by those enzymes. The cut fragment was subcloned into pcDNA3.1 (-) vector with a fragment including the coding region of YFP (Val2-Lys273) cut by BamHI and NotI. CHO-K1 cells were seeded on glasses and transfected with the plasmid DNAs for the channels and a transfection marker of YFP or α1A-AR-YFP by lipofectamine2000 (Thermo Fisher scientific-JP, Tokyo, Japan). Electrophysiological experiments were carried out 24−72 h after the transfection. Estradiol, estriol, estrone, testosterone, hydrocortisone and cholesterol were purchased from FUJIFILM Wako Pure Chemical Corporation (Osaka, Japan). Progesterone was obtained from Nacalai Tesque (Kyoto, Japan). Estradiol, estrone and estriol were solved in DMSO. Testosterone, hydrocortisone, progesterone and cholesterol were solved in ethanol. All reagents were prepared as high concentration stock solutions and stored at −20 degrees. The stock reagent solution was diluted at arbitrary concentrations for the electrophysiological experiments.

### Electrophysiology

The macroscopic whole cell current was recorded from the fluorescent CHO-K1 cell (expressing YFP or α1A-AR-YFP), using the whole cell patch clamp technique with Axopatch 200B amplifiers, Digidata 1550B and the pClamp 11 software (Molecular Devices, CA, USA), as previously described [7]. The bath solution was composed of (in mM) 140 NaCl, 1 CaCl_2_, 4 KCl, 0.3 MgCl_2_, 10 HEPES (pH 7.4 adjusted with NaOH). The pipette solution contained (in mM) 130 KCl, 5 Na_2_-ATP, 3 EGTA, 0.1 CaCl_2_, 4 MgCl_2_, 10 HEPES and 0.3 GTP (pH 7.3 adjusted with KOH). After the membrane rapture, the cell was held at -80mV and then the ramp pulse (from -120mV to +40mV for 0.4 sec) was repetitively applied (every 5 sec). For the estradiol inhibition and recovery speeds analysis, the ramp pulse written above was applied every 2 sec. In all current recordings, the reagents were applied by using a fast perfusion system (VC77SP, Warner Instruments, MA, USA) [7].

### Molecular docking

The chemical structure data for estradiol were sourced from PubChem (https://pubchem.ncbi.nlm.nih.gov/) [23] and stored in the mol2 file format. The three-dimensional structure of mouse THIK-1 was generated by using the Alphafold3 program [24]. The human THIK-1 structures were obtained from the Protein Data Bank (PDB) entries 9BSN and 9FT7 for wild type THIK-1 and 9C09 for mutant S136P [8,25]. The structure 9FT7 exhibited similarity to the mouse model [26]; RMSD vs mouse model was 0.444 Å for 9FT7, 0.798 Å for 9C09, 0.812 Å for 9BSN. SwissDock (http://www.swissdock.ch/) was utilized with the AutoDock-Vina plugin [27,28], employing a channel search volume of 32 x 32 x 25 Å. The center was positioned near the central and bottom regions of the selective filter. A docking result depicting estradiol bound to human THIK-1 is presented, featuring color annotations generated using UCSF Chimera X 1.71. [26].

### Analysis and statistics

The current amplitude at the membrane potential of 0 mV was measured from every trace recorded upon the ramp pulse protocol to minimize the fraction of non-selective leak current. The current amplitude was normalized by cell capacitance to exclude the influence of the cell volume. The current density before the application of steroid, arachidonic acid or norepinephrine (NE) was set as the basal current density (I_0_). The effects of steroids or α1A-AR-YFP stimulation on the THIK-1 channels were evaluated as the ratio of the current density after the application of steroids or NE to I_0_ (I_steroids_/I_0_ or I_NE_/I_0,_ respectively). The speeds of the estradiol-induced inhibition (τ_inhibit_) and recovery from inhibition (τ_rec_) were estimated by fitting the decreasing phase of the current after the estradiol application and the recovery phase after washout to single exponential curve (Clampfit 11.2, Molecular Devices, CA, USA). A concentration of half inhibition (IC_50_) was estimated by fitting the concentration-inhibition curve to Hill equation (Origin2016; OriginLab, MA, USA). All data are expressed as mean and the standard deviations (S.D.). Effects of steroids on channels were statistically analyzed by paired Student’s *t*-test (I_0_ vs. I_steroids_). In the studies of mutational effect, the statistical significance between two or more than two groups was estimated by unpaired Student’s *t*-test or by a one-way analysis of variance (ANOVA) followed by Tukey’s test, respectively. Values of p ≤ 0.05 were considered as statistically significant (***: p ≤ 0.001, **: 0.001 < p ≤ 0.01, *:0.01 < p ≤ 0.05, n.s.: p > 0.05).

## Results

### Effects of steroids on the THIK-1 channel

**Fig 1:**
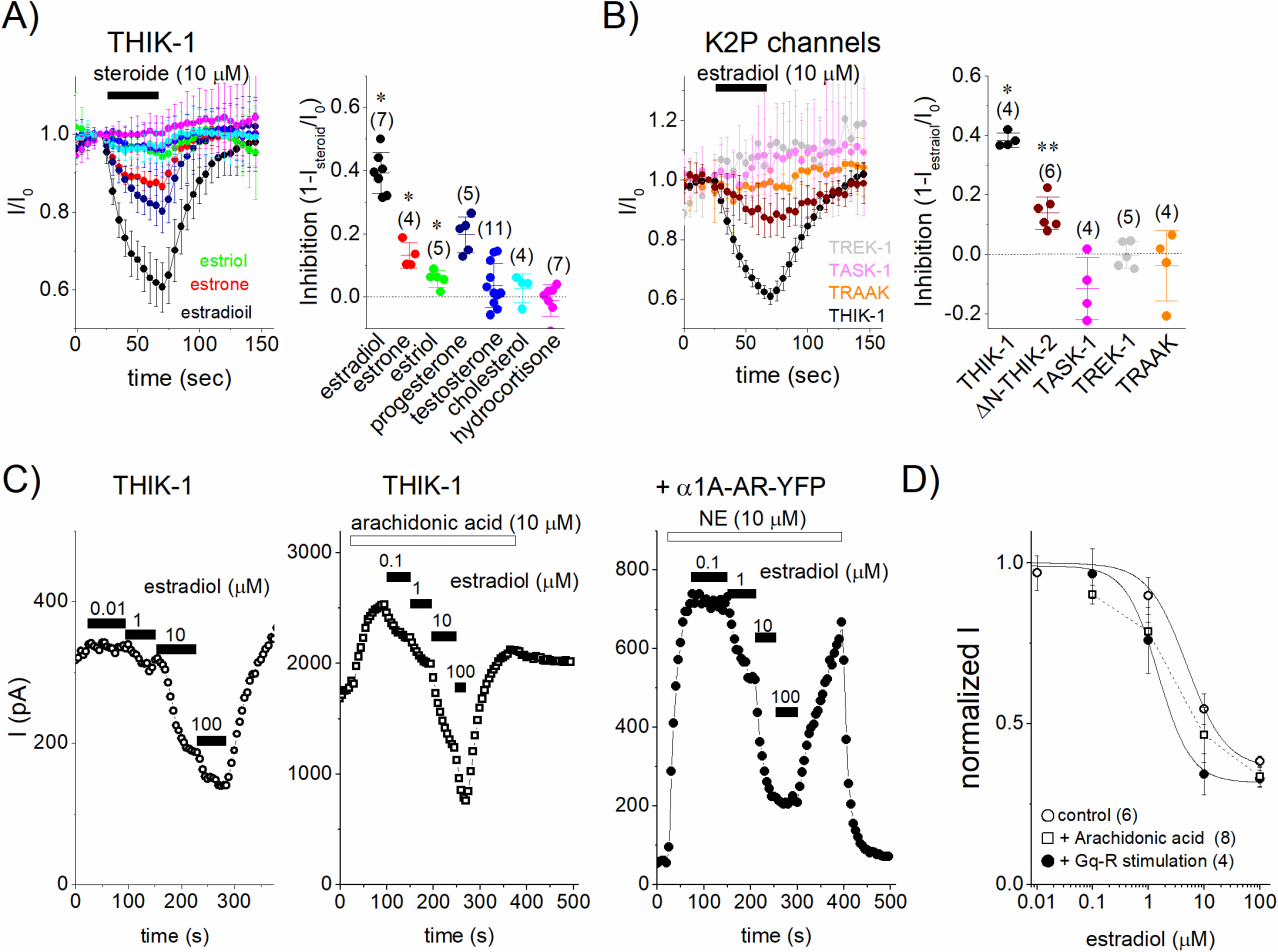
Inhibition of THIK-1 by estradiol. (A) Effect of steroids on THIK-1. The time-lapse changes in the normalized current amplitudes are shown (I/I_0_, left panel). Whole cell currents were recorded from CHO-K1 cells transfected with THIK-1 and transfection marker YFP. Ramp pulse from −120 mV to +40 mV (400 ms) was repetitively applied to cells every five seconds. Colored symbols show averages of the normalized amplitudes, matching the steroid labels by color. The black bar on the traces indicates when steroids (10 μM) were applied. The inhibition by steroids (1- I_steroid_/I_0_) is shown as circles and summarized in right panel. (B) Effect of estradiol on K2P channels. The time-lapse changes in the normalized current amplitudes are shown in the left panel. The black bar indicates the timing of estradiol (10 μM) application. The colored circles match the K2P labels by color. In the right panel, the circles indicate the inhibition (1- I_estradiol_/I_0_). (C) Estradiol concentration dependent inhibition of THIK-1. Time-lapse changes in the amplitudes of the THIK-1 channel currents, as written above. Symbols represent the current amplitudes at 0 mV and black bars indicate the application of the indicated concentration of estradiol. White bars indicate the application of arachidonic acid (10 μM, center panel) or NE (10 μM, right panel). (D) The concentration-inhibition relationship of THIK-1 by estradiol. Symbols represent the averages of the normalized amplitude in each combination, as indicated in the panel. Solid lines represent the curves obtained by fitting the plots to Hill equation. Bars represent the mean and S.D. The number of cells is indicated in parentheses. *: 0.01 < p ≤ 0.05, **: 0.001 < p ≤ 0.01 by paired Student’s *t*-test.

Initially, we assessed the impact of estrogen (estradiol, estrone, estriol), progesterone, testosterone, cholesterol and hydrocortisone on mouse THIK-1 channel activity, by calculating the steroid-induced changes in the current amplitudes recorded from CHO-K1 cells. Application of estradiol (10 μM) resulted in decreases in the current amplitude; this effect was reversed following washout (Fig 1A). Estradiol, estrone, estriol and progesterone showed current decreases (nearly 40%, 13%, 6% and 20% (p = 0.0503), respectively) (Fig 1A right panel). Application of solvents without steroids did not change the current amplitudes: I_DMSO_/I_0_ = 1.00 ± 0.05, n = 5 for 0.02% v/v DMSO and I_ethanol_/I_0_ = 0.99 ± 0.04, n = 7 for 0.05% v/v ethanol. These results demonstrate that estrogen, but not solvents, inhibits THIK-1. As THIK-1 was reported to be highly expressed in testis [3], testosterone was expected to modulate THIK-1 but did not exert significant effect at 10 μM (Fig 1A). Next, effect of estradiol was tested on other K2P channels, such as mouse THIK-2, mouse TREK-1, human TRAAK and mouse TASK-1 (Fig 1B). To increase the surface expression, N-terminal residues of THIK-2 were deleted (ΔN-THIK-2) [7,22]. Estradiol suppressed ΔN-THIK-2 but not TASK-1, TREK-1 and TRAAK, suggesting that estradiol’s inhibitory action is not broadly present among K2P channels.

The relationship between estradiol concentration and THIK-1 inhibition was investigated. The current amplitude of THIK-1 was decreased by the application of estradiol in a concentration dependent manner (Fig 1C, left panel). The effective concentration of estradiol was in the range of those reported in the studies of other K^+^ channels (Fig 1D, open circle, IC_50_ = 4.9 ± 1.5 μM, n = 6) [19–21], although IC_50_ is much higher than that to the G protein coupled estrogen receptor (GPER) [29,30]. As the sensitivity of the pore blocker tetrapentylammonium (TpenA) to THIK-1 was increased when the channel is activated [8], it was possible that the channel activation may increase sensitivity to estradiol. Application of arachidonic acid (10 μM) or NE (10 μM) to stimulate Gq-coupled α1A-AR increased the current amplitude (Fig 1C, center and right panels), as has been reported [3,7]. The NE effect was results of Gq-coupled α1A-AR stimulation, as amplitude remained unchanged after the NE application in cells expressing either α1A-AR-YFP (I_NE_/I_0_ = 1.00 ± 0.28, n = 5) or THIK-1 with YFP (I_NE_/I_0_ = 1.00 ± 0.04, n = 4). When THIK-1 was activated, it enhanced estradiol’s inhibitory effect in a distinct way (Fig 1D). Arachidonic acid sensitized THIK-1 to 0.1 μM estradiol, which made the estimation of IC_50_ difficult (Fig 1D dotted line). The THIK-1 activation by the Gq-R stimulation parallelly shifted the concentration-inhibition curve leftwards (IC_50_ = 1.5 ± 0.6 μM, n = 4, Fig 1D). THIK-1 activation slightly increased sensitivity to estradiol, but not enough for suppression at physiological concentrations [31].

### Candidates of estradiol docking site

**Fig 2:**
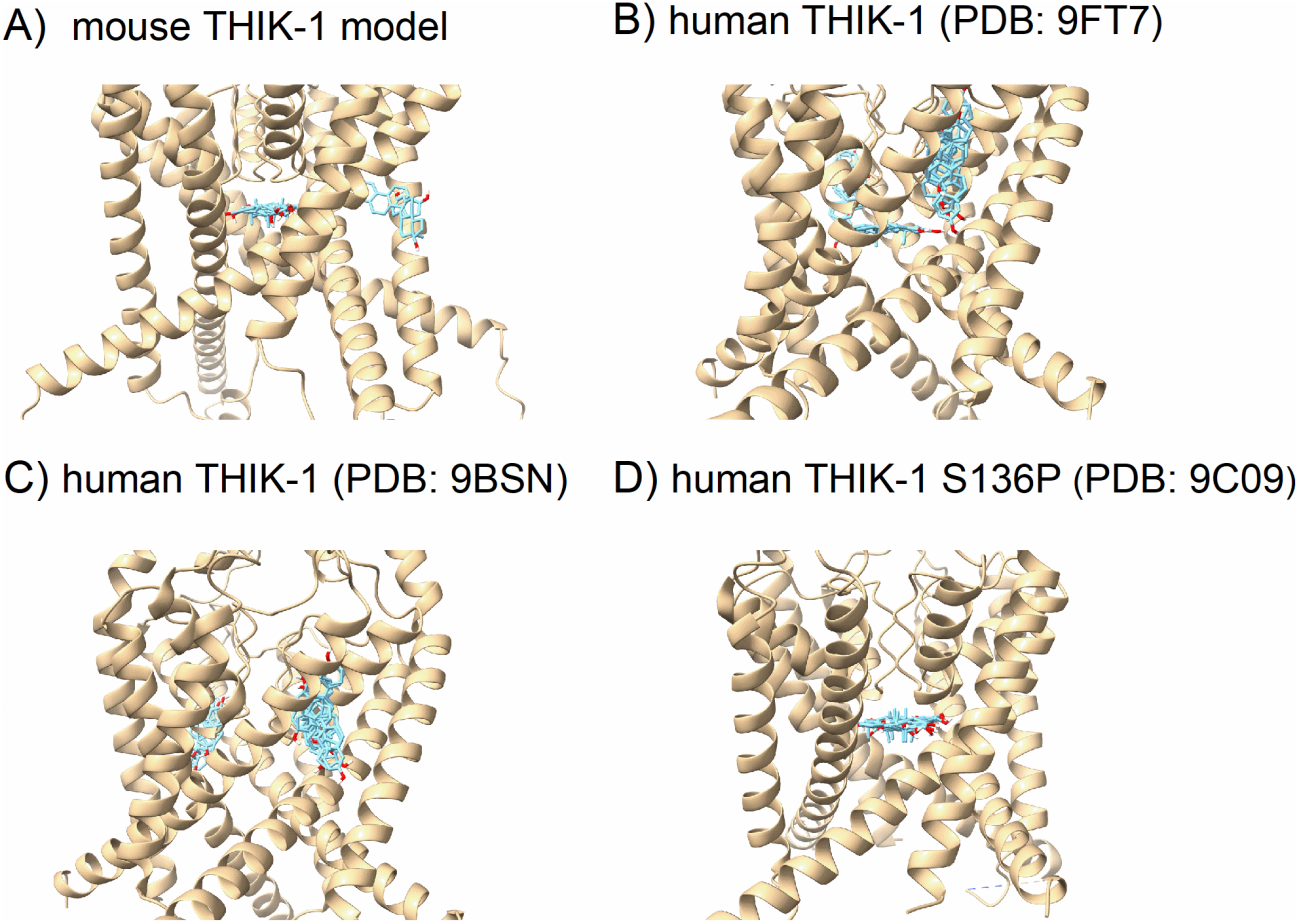
Potential estradiol docking sites for mouse THIK-1 model and human THIK-1. (A-D) Structures of the estradiol docked THIK-1. Shown are the structures of estradiol docked mouse THIK-1 model, human THIK-1 (PDB: 9FT7, 9BSN) and its mutant S136P (PDB: 9C09) (top 10−12 poses). Estradiol is depicted in cyan, and its hydroxy group is shown in red and white. (A) The estradiol docked mouse THIK-1 model. Affinity results (kcal/mole): for the upper cavity (pond), values range from −8.97 to −5.98 (8 poses); for lower part of the K2P modulator pocket, −6.70 and −6.29 (2 poses). (B) The estradiol docked human THIK-1 (PDB: 9FT7). Affinity results (kcal/mole): for the K2P modulator pocket, values are −8.77 and −7.20 (10 poses); for the pond, −7.07 and −7.03 (2 poses). (C) The estradiol docked human THIK-1 (PDB: 9BSN). Affinity results (kcal/mole): for the K2P modulator pocket, values range from −9.24 to −6.51 (11 poses). (D) The estradiol docked human THIK-1 mutant S136P (PDB: 9C09). Affinity results (kcal/mole): for the pond, values range from −9.93 to −7.10 (11 poses).

Steroids are known to disturb membrane fluidity [32], which may result in THIK-1 inhibition. However, this was not likely, since effects of estradiol and progesterone have been reported to be opposite on the fluidity [32,33]. In addition, estradiol did not influence the TRAAK activity which couples to lipid interaction [34]. Therefore, estradiol was thought to directly inhibit THIK-1. Then we investigated docking sites of estradiol, by utilizing the molecular protein docking simulation (SwissDock, as described in Experimental procedures) and mutagenesis. The results of the simulation exhibited two distinct estradiol-docking sites for tested THIK-1 structures (mouse THIK-1 model, human THIIK-1 (PDB: 9BSN and 9FT7) and S136P THIK-1 (PDB: 9C09)) [8,25] (Fig 2): the K2P modulator pocket (the site of polyunsaturated fatty acid interaction) and the upper cavity from the bottom of selective filter to the Y gate (pond) [8,25,35]. Therefore, we selcted the K2P modulator pocket and pond as possible estradiol docking sites.

### Alanine scanning for the residues of estradiol-mediated inhibition

**Fig 3:**
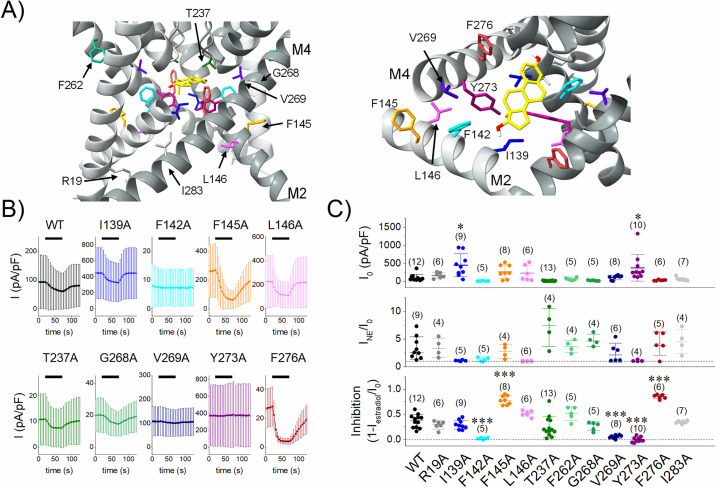
The alanine scanning for the investigation of the estradiol docking sites. (A) The structure of the estradiol docked THIK-1. Estradiol is depicted in yellow, and its hydroxy group is shown in red and white. The alanine introduced residues of human THIK-1 (PDB: 9FT7) are colored and indicated in left panel (side view) and the pond sites from diagonal top view are highlighted on the right panel. (B) Time-lapse changes in the current amplitudes of wild type and mutant THIK-1 channels. Ramp pulse from −120 mV to +40 mV (400 ms) was repetitively applied to cells every five seconds. Symbols represent the averages of the current densities at 0 mV and black bars indicate the application timing of estradiol (10 μM). The tested THIK-1 constructs are indicated above the traces. (C) The properties of wild type and mutant THIK-1. Circles represent the basal density (I_0_, upper panel), the Gq-R response (I_NE_/I_0_, middle panel) and the inhibitory effect of estradiol (1-I_estradiol_/I_0_, lower panel). Bars indicate means and S.D. The number of cells is indicated in parentheses. *: 0.01 < p ≤ 0.05, ***: p ≤ 0.001 vs. WT by one-way ANOVA followed by Tukey’s test.

Next, alanine mutation was introduced into residues at the K2P modulator pocket and pond (Fig 3A). Phe262 was an alanine introduced residue, because it is highly conserved through K2P. Nine residues at and around the pond, including Thr237 adjacent to the selectivity filter were also selected (Fig 3A). Due to the high conservation of amino acid sequences in THIK-1, the designated residue position and corresponding amino acid in mouse THIK-1 are consistent with those in human THIK-1, except for residue 136. Arg19 and Iso283 at the lower sites of the first and fourth TM domains (M1 and M4, respectively) were also tested (Fig 3A left), since they correspond to the residues of TASK-1 which are important for the interaction with cholesterol (Arg7 and Phe246) [16]. Alanine scanning examination revelated that F142A, V269A and Y273A failed to respond estradiol but the F145A and F276A potentiated estradiol’s inhibitory effect (Fig 3B, 3C bottom). The Y273A mutation resulted in significant increase in the basal current density (I_0_) and attenuated the Gq-R response (I_NE_/I_0_ < 1.2) (Fig 3C, top and middle panels), as recently reported [8]. Similarly, I139A showed increased basal density and the attenuation of Gq-R response but not of estradiol inhibition. The current increases and disengagement of the Gq-R dependent regulation, observed in the I139A and Y273 mutants, were thought to be caused by the lack or loosening of the Y gate [8,25]. Regarding the THIK-1 inhibition by estradiol, the I139A mutation did not enhance the estradiol affinity (IC_50_ = 13.7 ± 6.6 μM, n = 4) and Y273A eliminated the inhibition. Results of these mutations are contrast to TpenA sensitization [8], suggesting the difference in the inhibitory mechanism between estradiol and TpenA. The lack of estradiol inhibition in F142A might be due to the less activity of F142A: I_0_ = 7.5 ± 6.7 pA/pF for F142A (n = 5) and 92.0 ± 92.7 pA/pF for wild type (n = 12). Interestingly, the V269A mutation attenuated estradiol inhibition without changing the density and the Gq-response (Fig 3C), indicating that Val269 plays important role in estradiol-mediated inhibition but not in channel activation. In contrast to the impairment of estradiol inhibition, the F145A and F276A mutations significantly potentiated the inhibitory effect (Fig 3B, 3C). The channel properties were not significantly changed by these mutations, although the basal density of F276A appeared smaller than that of WT (I_0_ = 30.6 ± 13.5 pA/pF, n = 6). Taken together, mutagenesis analyses have identified Phe142, Val269, and Tyr273 as critical residues for estradiol-mediated inhibition and have revealed F145A and F276A as the mutations for estradiol sensitization.

### Critical residues for the estradiol-mediated inhibition

**Fig 4:**
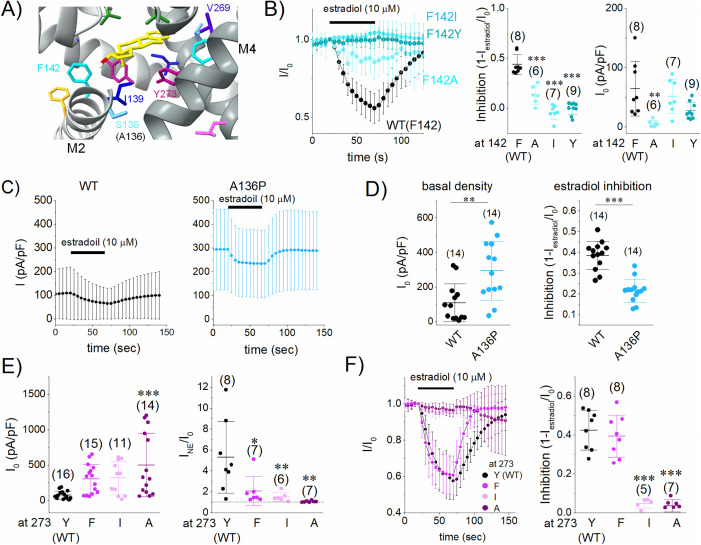
Contributions of Phe142 and Tyr273 to inhibitory effect of estradiol on THIK-1. (A) The structure of the estradiol docked THIK-1. Estradiol is depicted in yellow, and its hydroxy group is shown in red and white. Ser136 (mouse Ala136), Ile139, Phe142, Val269 and Tyr273 of human THIK-1 (PDF: 9FT7) are shown in cyan, blue, aqua, indigo and violet, respectively. (B) Effect of estradiol on the Phe142 mutants. The time-lapse changes of the normalized current amplitudes are shown in left panel. Symbols represent the averages of the normalized amplitudes, and the black bar indicates the timing of estradiol (10 μM) application. In the center and right panels, circles represent the inhibitory effect of estradiol (1-I_estradiol_/I_0_, center panel) and the basal current density (I_0_, right panel), respectively. (C) Effect of estradiol on WT and the A136P mutant THIK-1. The time-lapse changes of the normalized current amplitudes are shown. Symbols and bars are described above. (D) Properties of the A136P mutant. Circles represent the current density (I_0_, left panel) and estradiol inhibition (1-I_estradiol_/I_0_, right panel). (E) Properties of the Tyr273 mutants. Circles represent the current density (I_0_, left panel) and the Gq-R response (I_NE_/I_0_, right panel). (F) Effect of estradiol on the Tyr273 mutants. Shown in left is the time-lapse changes in the averages of the normalized current amplitudes. In right panel, circles represent the inhibitory effect of estradiol (1-I_estradiol_/I_0_). Bars indicate means and S.D. The number of cells is indicated in parentheses. *: 0.01 < p ≤ 0.05, **: 0.001 < p ≤ 0.01, ***: p ≤ 0.001 vs WT by one-way ANOVA followed by Tukey’s test (B, E, F) or by unpaired *t*-test (D).

Phe142, Val269 and Tyr273 face into the pond of the THIK-1 cavity (Fig 4A) and their alanine mutations attenuated estradiol inhibition (Fig 3C). The insensitivity to estradiol may be attributed to the less activity of F142A and the excess activity of Y273A, which could potentially be outside of channel regulation [8]. The docking simulation indicated a potential interaction between estradiol and the part of their benzene rings (Phe142 and Tyr273). Consequently, further mutations were introduced at these specific residues. The F142Y and F142I mutations eliminated estradiol inhibition without suppressing basal activity (Fig 4B). These mutants responded to Gq-R: I_NE_/I_0_ = 3.5 ± 1.6 (n = 4) for F142I and 13.8 ± 7.0 (n = 4) for F142Y. Based on these findings, phenylalanine, but not benzene ring, at position 142 appears to be indispensable for the estradiol-mediated inhibition. The structural analyses have revealed that Tyr273 faces each other and forms the narrowest part of the permeation pathway [8,25]. The Y273A mutation is thought to widen and/or disrupt the narrow Y gate and loses the benzene ring. As for the widening Y gate, the S136P mutation in human THIK-1 is shown to increase distance between Tyr273 residues and current density (PDB: 9C09) [25]. The corresponding mutation of mouse THIK-1, A136P, increased density and attenuated estradiol inhibition (Fig 4C, 4D), without impacting Gq-R response (I_NE_/I_0_ = 2.24 ± 1.27, n = 5). Based on the findings from the Y gate widening mutant, tyrosine at position 273 is considered essential for the inhibitory effect of estradiol. Then we investigated the effects of benzene ring at residue 273, by examining the effects of the Y273I and Y273F mutations. There is a trend that the basal current density increased along with the decrement of the side chain volume while the Gq-R response was not eliminated by those mutations except for Y273A (Fig 4E). The estradiol inhibition was maintained in the Y273F mutation but not the Y273I one (Fig 4F), indicating that benzene ring at residue 273 is necessary for the estradiol-mediated inhibition. Overall, Phe142, V269 and Tyr273 are critical for the inhibitory effect of estradiol on THIK-1.

### Potentiation of the inhibitory effect of estradiol on THIK-1

**Fig 5:**
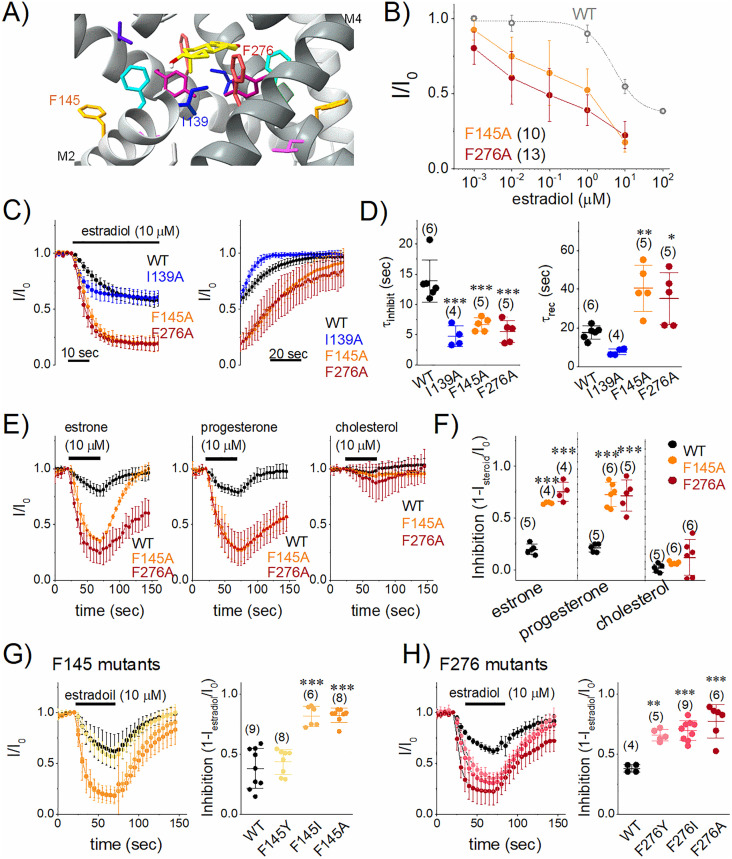
Potentiation of the inhibitory effect of estradiol by the F145A and F276A mutations. (A) The structure of estradiol docked THIK-1. Estradiol is depicted in yellow, and its hydroxy group is shown in red and white. Ile139, Phe142, Phe145, Tyr273 and Phe276 of human THIK-1 (PFB, 9FT7) are colored in blue, aqua, orange, violet and dark brown, respectively. (B) The concentration-inhibition relationships of F145A and F276A by estradiol. The dashed black line, shown for comparison, is identical to the curve of wild type THIK-1 in figure 1. (C) Speed of estradiol inhibition and recovery. Ramp pulse from -120 mV to +40 mV (400 ms) was repetitively applied to cells every two sec. Symbols represent the averages of the normalized amplitudes (I/I_0_) in the indicated construct; the current decrease phase (left) and the recovery phase (right). (D) Summary of the inhibition and recovery speed. The rates of inhibition and recovery kinetics were estimated by fitting the current decrease and increase to a single exponential curve, respectively. Each symbol represents rates of the inhibition (τ_inhibit_, left) and recover (τ_rec_, right). (E) Effect of steroids on the F145 and F276 mutants. The time-lapse changes in the normalized current amplitudes of the indicated THIK-1 channels. Symbols represent the averages of the normalized amplitudes at each time point. The timing of steroids application is shown by black bar on the trace. (F) Summary of THIK-1 inhibition by steroids. Circles represent the inhibitory effect of steroids (1-I_steroids_/I_0_). (G and H) Characterization of Phe145 and Phe276 mutants. The time-lapse changes in the normalized current amplitudes of the F145 and F276 mutants are shown in left panels of G and H, respectively. Summary of estradiol inhibition for F145 and F276 mutants are shown in right panels of G and H, respectively. Circles represent the inhibitory effect of estradiol (1-I_estradiol_/I_0_). Bars indicate means and S.D. The number of cells is indicated in parentheses. *: 0.01<p≤ 0.05, **:0.001< p≤ 0.01, ***: p≤ 0.001 vs. WT by one-way ANOVA followed by Tukey’s test.

The alanine scanning revealed that the F145A and F276A mutations potentiated the inhibitory effect of estradiol without altering the basal density and the Gq-R response (Fig 3C). Docking simulation results indicate that Phe145 does not directly interact with estradiol (Fig 5A), implying the mutation may cause rearrangements of the TM domains and/or changes in the pond conformation. The replacement of phenylalanine with alanine at residue 145 or 276 caused the sensitization to estradiol: these mutations largely shifted the concentration-inhibition curves leftward (Fig 5B). In these mutants, as well as I139A which accelerated the TpenA inhibition [8], the estradiol inhibition was observed to occur at a faster rate compared to the wild type (Fig 5C, 5D). As the A136P also accelerated the inhibition speed (τ_inhibit_ = 4.8 ± 1.7 sec, n = 4), the F145A and F276A mutations were interpreted to increase accessibility of estradiol to its docking sites. In contrast to the inhibition speed, recovery upon washout of estradiol was decelerated in F145A and F276A (Fig 5C, 5D), which may contribute to the observed increase in affinity. The F145A and F276A mutations also enhanced the inhibitory effect of estrone and progesterone, but not cholesterol (Fig 5E, 5F). Similar enhancement was observed when estriol was applied (by approximately 30% decrease in F145A and F276A, vs. 5% in wild type). Based on these findings, it was hypothesized that such mutations induce conformational change in the docking site that allows greater accessibility for multiple steroid ligands or potentially create an alternative docking site.

The benzene ring at residue 273 plays a crucial role in estradiol-mediated inhibition (Fig 4F), raising a speculation that the benzene rings located at residues 145 and 276 contribute significantly to inhibition of estradiol inhibition. Then Tyr or Ile mutation was introduced into the residue of Phe145 or Phe276. The F to I mutations exhibited the enhancement of estradiol inhibition (Fig 5G, 5H). While the F145Y mutation did not enhance estradiol inhibition, the F276Y mutation resulted in a notable increase (Fig 5G, 5H). These findings suggest that the lack of benzene ring at residue 145 sensitizes estradiol, whereas the lack of phenylalanine at residue 276 causes estradiol sensitization. The significance of phenylalanine at 276 was further confirmed by findings that the basal densities of F276I and F276Y were similar to those of F276A (approximately 25 pA/pF).

### The effect of estradiol on the THIK-1 variants

**Fig 6:**
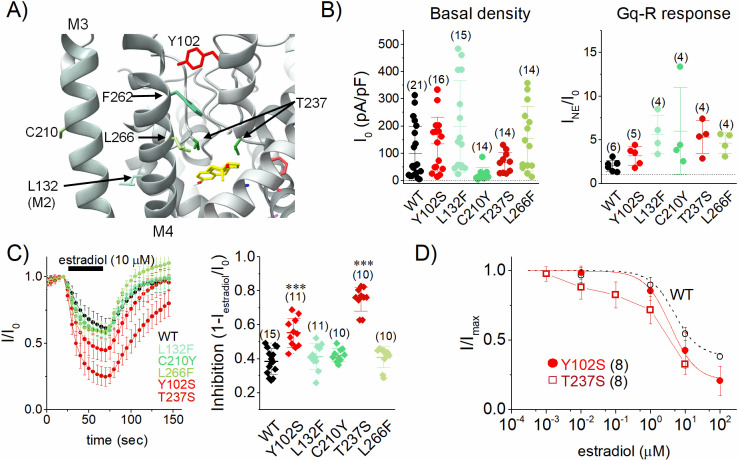
Effects of estradiol on the THIK-1 variants. (A) The structure of estradiol bound THIK-1. Estradiol is depicted in yellow, and its hydroxy group is shown in red and white. Tyr102, Leu132, Cys210, Thr237, Phe262 and Leu266 of human THIK-1 (PFB, 9FT7) are colored and indicated. (B) Properties of the mouse THIK-1 mutants with reported variation in human THIK-1. Circles represent the current density (I_0_, left panel) and the Gq-R response (I_NE_/I_0_, right panel). (C) Effect of estradiol on the mouse THIK-1 mutants with reported variation in human THIK-1. The time-lapse changes in the normalized current amplitudes of the indicated mutants are shown in the left panel. Symbols represent the averages of the normalized current amplitudes and the black bar on the traces indicates the application timing of estradiol. Summary of estradiol inhibition is shown in the right panel. Circles represent inhibitory effect of estradiol on the indicated variants (1-I_estradiol_/I_0_). (D) Concentration-inhibition relationships of the Y102S and T237S mutations by estradiol. Symbols and thin bars show means and standard deviations. Solid red line obtained fitting to Hill equation for Y102S variant. The dashed black line, shown for comparison, is identical to the curve of wild type THIK-1 shown in figure 1. Correlation between estradiol concentration and T237S inhibition could not be fitted by Hill equation. Bars represent the means and S.D. The number of cells is indicated in parentheses. ***: p ≤ 0.001 vs WT by one-way ANOVA followed by Tukey’s test.

A low concentration of estradiol (10 nM) reduced the activity of the F276A mutant by approximately 40% (Fig 5B); this concentration falls within the physiological range [31]. It was possible that certain variants of human THIK-1 might increase the sensitivity to estradiol. To teste this possibility, we referred the human THIK-1 variants to database of ClinVar (https://www.ncbi.nlm.nih.gov/clinvar/) and dbSNP (https://www.ncbi.nlm.nih.gov/snp/). The mutation which is detected on the TM domains in ClinVar was introduced into mouse THIK-1 channel (Y102S, L132F, C210Y and L266F shown in Fig 6A). A variant at residue 273, T237S, was also introduced into mouse THIK-1 (dbSNP, rs2503855259), since estradiol-mediated inhibition varied in T237A (Fig 3C) and Thr237 faces the pond (Fig 6A). All mutations did not change the basal density and Gq-R response (Fig 6B), although basal densities of L132F and C210Y appeared large and small, respectively. Regarding estradiol effect, Y102S at the K2P modulator pocket and T237S significantly enhanced estradiol-mediated inhibition (Fig 6C). The affinity of estradiol to Y102S was almost identical to that of wild type (IC_50_ = 3.8 ± 0.7 μM, n = 4, Fig 6D). Mutations at the K2P modulation site (Y102S, F262A, L266F) did not attenuate estradiol inhibition, indicating this site is not involved in inhibition mechanisms of estradiol. T237S was sensitized to estradiol: the current decrease was observed at 10 nM estradiol (Fig 6D). Recovery from estradiol inhibition in T237S was measured to be slow (τ_rec_ = 42.6 ± 12.0 sec, n = 5)(cf. Fig 5D). T237S also enhanced the estrone and estriol inhibition; inhibition was 39.0 ± 13.6% (10 μM estrone, n = 5) and 17.9 ± 6.1% (10 μM estriol, n = 4)(cf. Fig 1A). The effects of the T237S mutation were similar to those of the F145A and F276A mutations and different from those of the T237A mutation. The observed variation in estradiol-mediated inhibition between T273A and T273S suggests that minor alterations at residue 237, which is linked to the selective filter, can influence estradiol inhibition.

## Discussion

This study demonstrated that estradiol inhibits the THIK-1 channel and that Phe142, Val269, and Tyr273, which are involved in pond conformation, are requisite for estradiol-mediated inhibition. In addition, mutations F145A, F276A, and T237S were shown to increase the sensitivity of THIK-1 to estradiol.

### The critical domains for estradiol-mediated inhibition

Residues including Ile139, Phe142, Thr237, Val269, and Tyr273 are integral to shaping the pond conformation and contribute to the inhibitory actions of TpenA and potentially anesthetics [8]. Docking simulations suggest that estradiol may reside within the THIK-1 pond (Figs 2 and 3A), and estradiol-mediated inhibition was eliminated by F142A, V269A, or Y273A mutations (Fig 3). The F142A and Y273A mutations, unlike V269A, altered both basal activity and Gq-R responses (Fig 3). Conversely, F142I and Y273I mutations abolished estradiol’s inhibitory effect while maintaining unchanged basal activity and Gq-R response (Fig 4). These findings support the hypothesis that estradiol interacts with residues that affect pond conformation, resulting in THIK-1 inhibition. However, docking simulation results do not confirm direct interaction between estradiol and THIK-1. Although the K2P modulator pocket was provided as a docking candidate for different THIK-1 structures (Fig 2), the F262A, Y102C and L266F mutations did not attenuate estradiol-mediated inhibition (Figs 3C, 6C). Two pond mutants, I139A and T237A, were inhibited by estradiol (Fig 3), while T237S inhibited by 10 nM estradiol (Fig 6). Importantly, mutational effects on inhibitory action varied among TpenA, anesthetics, and estradiol: I139A and Y273A have correlated with enhanced sensitivity to TpenA, whereas T237A reduced inhibition by TpenA and anesthetics [8]. These differences in mutational impact indicate that estradiol exerts an inhibitory mechanism differing from those of TpenA and anesthetics. Additionally, estradiol’s inhibitory effects differed between T237A and T237S. Since Thr237 is adjacent to the GYG motif, subtle environmental alterations around this residue may affect ion permeability. Collectively, these observations demonstrate that residues contributing to pond conformation are essential for estradiol-mediated inhibition, although the definitive docking site for estradiol remains unresolved. It is suggested that estradiol causes conformational changes in the THIK-1 pond, which leads to a reduction in K^+^ permeation.

The I139A and A136P mutations did not abolish estradiol inhibition, yet only A136P not I139A attenuated this inhibitory effect. As for the distinct outcomes with estradiol inhibition, these could arise from variations in Y gate arrangements: I139A might eliminate bulky groups at the gate, whereas A136P may increase the distance between Tyr273 residues [8,25]. Notably, I139A impaired the Gq-R response (Fig 3C), but A136P preserved it (I_NE_/I_0_ = 2.2 ± 1.3, n = 5). These findings suggest that regulatory and inhibitory mechanisms of THIK-1 are complex and cannot be explained simply. The same complexity applies to the influence of THIK-1 activation on estradiol-mediated inhibition (Fig 1D). There is no direct correlation between the degree of K^+^ permeation and the extent of inhibition by estradiol. The Gq-R stimulation increased current amplitudes (I_NE_/I_0_ = 6.2 ± 4.3, n = 4) and estradiol affinity, while F145A and F276A intensified estradiol-mediated inhibition without boosting basal activity. This study does not fully resolve the details of estradiol’s inhibitory mechanism; future structural research will be necessary to elucidate these processes.

### Enhancement of the estradiol-mediated THIK-1 inhibition

The F145A, F276A, and I139A mutations led to faster estradiol inhibition (Fig 5), indicating these changes may facilitate conformational shifts that either improve estradiol access or quicken the transition from a normal to a less permeable state. Both F145A and F276A, as well as T273S, showed slow recovery from estradiol inhibition, which suggests these mutations may increase ligand affinity. Additionally, these variants enhanced estradiol inhibition (Figs 5, 6) and heightened the inhibitory effects of estrone, estriol, and progesterone. Since the mutations are located at different positions (Phe145 on M2, Thr237 before the selectivity filter, and Phe276 on M4) and residues 145 and 276 are distant from the pore, they may promote a THIK-1 conformation that is generally more receptive to various steroid ligands (such as estrogen and progesterone), rather than specifically boosting estradiol binding. However, the exact structural alterations induced by these mutations in the TM domain are still unclear and require further studies.

The physiological concentration of estradiol was reported from 0.04 to 1.3 nM and to reach 25 nM during pregnancy [31,36]. Neural estradiol concentration was also reported from 0.05 to 12 nM [37]. Physiological concentration of estradiol is insufficient to suppress THIK-1, suggesting that estradiol-mediated inhibition is of limited significance. THIK-1 variants which can be inhibited by low concentration of estradiol, such as T237S, may mediate estrogen’s inhibitory effects in relevant physiological or pathophysiological contexts.

In conclusion, estradiol inhibits the THIK-1 channel, which requires residues Phe142, Val269, and Tyr273 located in the pond of THIK-1. The inhibition is potentiated by the F145A, F276A and T237S mutations. The inhibitory effect of estradiol may have physiologically or pathologically significance for THIK-1 variants that are inhibited by low concentration of estradiol.
